# A deep dive into hyperbaric environments and intraocular pressure—a systematic review

**DOI:** 10.3389/fmed.2024.1365259

**Published:** 2024-04-03

**Authors:** Paul Connor Lentz, Sheng Yang Lim, Bjorn Kaijun Betzler, Darby D. Miller, Syril K. Dorairaj, Bryan Chin Hou Ang

**Affiliations:** ^1^Mayo Clinic Alix School of Medicine, Jacksonville, FL, United States; ^2^Department of Ophthalmology, Tan Tock Seng Hospital, National Healthcare Group Eye Institute, Singapore, Singapore; ^3^Department of Ophthalmology, Mayo Clinic, Jacksonville, FL, United States; ^4^Department of Ophthalmology, Woodlands Health Campus, National Healthcare Group Eye Institute, Singapore, Singapore

**Keywords:** intraocular pressure, glaucoma, diving, atmospheric pressure, hyperbaric

## Abstract

**Purpose:**

SCUBA diving exposes participants to a unique hyperbaric environment, but few studies have examined the effects of such an environment on intraocular pressure (IOP) and glaucoma. This systematic review aims to consolidate recent literature findings regarding the impact of increased atmospheric pressure on IOP and glaucoma.

**Methods:**

Three online databases were searched to identify publications encompassing the subjects of diving or increased atmospheric pressure in conjunction with IOP or glaucoma. Three reviewers independently screened the publications and identified eligible articles. Relevant data was extracted from each article. The heterogeneity of the data precluded the conduct of a meta-analysis.

**Results:**

Nine studies met the inclusion criteria. Six experimental studies employed hyperbaric chambers to measure IOP under simulated diving conditions. Among these, IOP exhibited a reduction with increased atmospheric pressures in four studies, while the findings of two studies were inconclusive. One study measured IOP pre- and post-dive and another measured IOP with and without a diving mask. Post-dive, a decrease in IOP was observed, and a statistically significant reduction was noted when subjects wore a diving mask. A retrospective study examining the incidence of acute angle closure glaucoma attack found no association with weather or atmospheric pressure.

**Conclusion:**

The majority of studies found IOP to decrease with increased atmospheric pressure and after diving. The mechanisms underlying this reduction remain incompletely understood, with potential contributors including changes in ocular blood flow, sympathetic responses, and increased oxygenation. Hyperbaric chambers may have potential in future glaucoma treatments, but more studies are required to draw reliable conclusions regarding the safety of diving for glaucoma patients.

## Introduction

In recent years, SCUBA diving has seen significant growth in global popularity as both a professional and recreational activity (1, 2). While relatively safe with appropriate prerequisite training and certification, diving is still associated with potential risks. Divers are exposed to a hyperbaric environment, defined as an environment with increased atmospheric pressure, which may have diverse effects on the body, potentially exacerbating various medical conditions (3). Consequently, SCUBA diving presents several contraindications, including cardiac, pulmonary, otolaryngic, and neurologic diseases (4).

The effects of SCUBA diving on the eye have also been reported. These include conditions such as ocular barotrauma, caused by a pressure differential between the interior and exterior of the diving mask (mask squeeze) (5–7), ophthalmic manifestations of decompression sickness (8, 9) such as retinal vein occlusion and choroidal ischemia, as well as other ophthalmic manifestations, including nystagmus, diplopia, optic neuropathy, and visual field defects (10).

While the effects of diving on human physiology (11) have been well reported, its ocular manifestations, particularly on intraocular pressure (IOP), have been less studied. The relationship between diving and IOP can be important in patients with glaucoma, as IOP remains a major modifiable risk factor for the disease and has been shown to significantly influence disease progression. Glaucoma also remains the leading cause of irreversible blindness globally, with its prevalence estimated to be as high as 3.5% of adults, between the ages of 40 to 80 years old (12).

The hyperbaric environment (13) experienced during diving may affect IOP by various mechanisms, including peripheral vasoconstriction, as well as increased blood pressure and ocular perfusion pressure (14, 15). Besides high atmospheric pressures, several other factors experienced during diving, such as cold temperatures (16) and increased oxygen concentration (17–20), may also affect IOP.

Hence, this systematic review aims to summarize the current literature to provide a comprehensive overview of the effects of diving and hyperbaric environment on IOP.

## Methods

**Figure 1:**
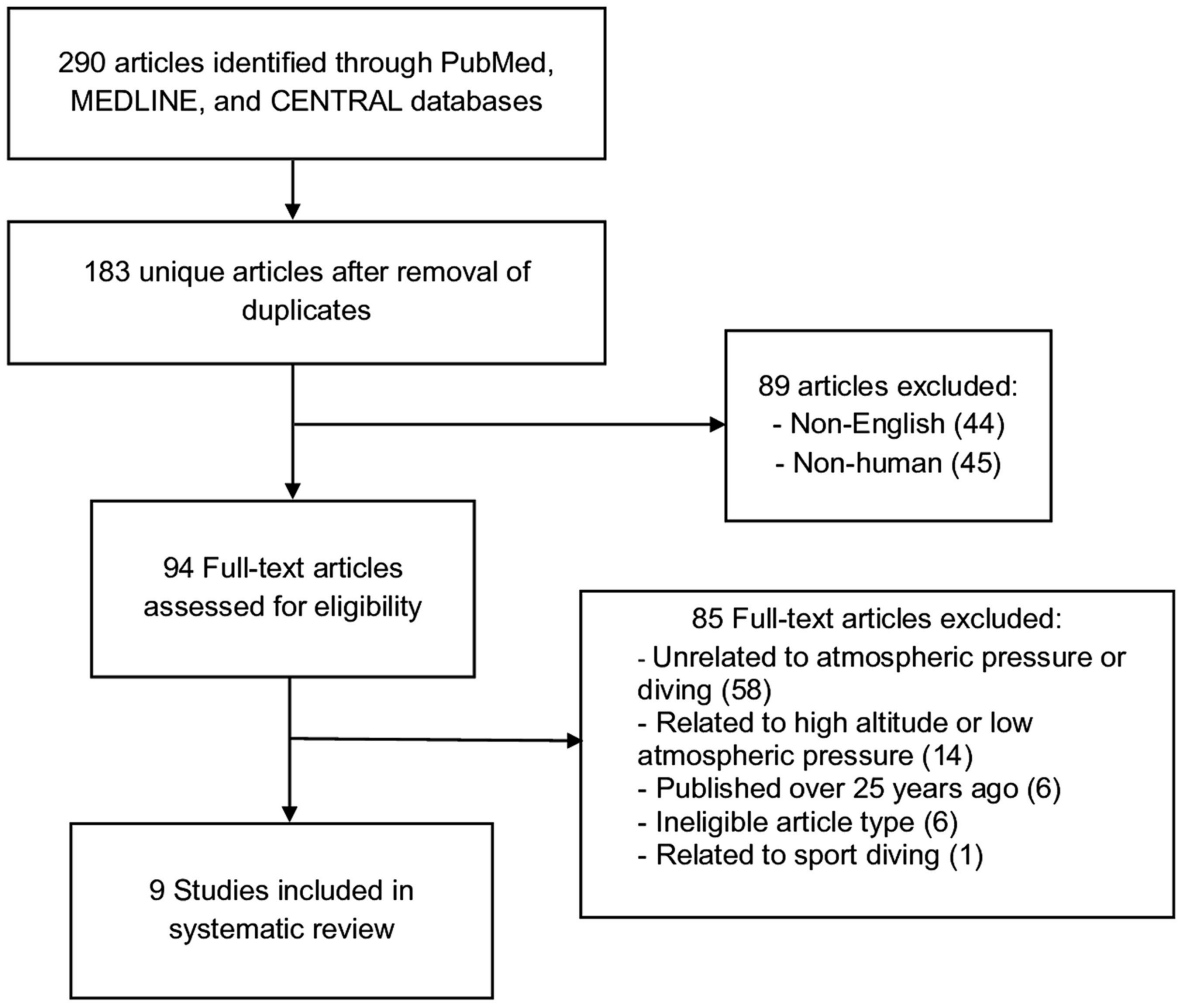
PRISMA flowchart.

This systematic review was conducted in accordance with the guidelines stated in the Preferred Reporting Items for Systematic Reviews and Meta-Analyses (PRISMA) (21). An electronic search of the PubMed, Medline, and CENTRAL databases was performed from the date of database inception until July 1, 2023, as illustrated in Figure 1.

We limited our literature search to studies that explored the relationship between diving or increased atmospheric pressure, with IOP or glaucoma. A combination of subject headings and text headings were used to define search terms as needed. Our search included the terms “(Intraocular Pressure OR Glaucoma) AND (Diving OR Underwater OR Atmospheric Pressure OR Hyperbaric) NOT Oxygen.” The decision was made to exclude the term “oxygen” from the search criteria to mitigate potential confounding factors, such as the inclusion of studies related to hyperbaric oxygen therapy. A secondary search was conducted by reviewing the references of all retrieved articles and relevant review articles to identify any additional potentially pertinent studies.

Three authors (PCL, LSY, BBK) independently assessed studies to determine eligibility for inclusion. In the event of discrepancies, differences were resolved by a senior author (SD, BCHA). Non-English articles and those that did not involve human participants were excluded from this review. Full text articles were assessed for eligibility. Papers unrelated to atmospheric pressure or diving, with a primary focus of high altitude or hypobaric environments, and those published over 25 years ago, were excluded from analysis. Ineligible article types included case reports, reviews, and letters to the editor. One report was excluded as it focused on sport diving.

A risk of bias assessment was performed by two authors (PCL, SYM) on applicable studies with the Risk of Bias in Non-Randomized Studies—of Intervention (ROBINS-I) tool (see Table 1) (22). Discrepancies in assessment were resolved through discussion until a consensus was reached.

**Table 1:** Evaluation of evidence quality—ROBINS-I tool (22).

	Confounding^a^	Selection of participants	Classification of interventions	Deviations from intended interventions	Missing data	Measurements of outcomes	Selection of reported results	Overall ROB judgment
Deleu 2022	Low	Moderate	Low	Low	Low	Moderate	Low	Moderate
Mazo 2023	Low	Moderate	Low	Low	Low	Moderate	Low	Moderate
Van de Veire 2008	Low	Moderate	Low	Low	Moderate	Low	Low	Moderate
Vercellin 2021	Low	Moderate	Low	Low	Low	Low	Low	Moderate
Albis-Donado 2020	Low	Moderate	Low	Low	Low	Moderate	Low	Moderate
Huang 2002	Low	Low	Low	Low	Low	Moderate	Low	Moderate
Peters 1999	Low	Low	Low	Low	Low	Moderate	Low	Moderate
Goenadi 2016	Low	Moderate	Low	Low	Low	Moderate	Low	Moderate
Bojić 2001^b^	Low	NA	NA	NA	NA	NA	NA	NA

^a^Confounding domain not applicable to before-after studies. ^b^Robins-I Tool not applicable to retrospective observational studies. ROB, Risk of Bias.

## Results

### Study characteristics

Table 2 provides a summary of the key characteristics of the included studies. Two hundred and ninety studies were identified of which nine were eventually included. Six of the nine included studies focused on measuring IOP within hyperbaric chambers to simulate the increased atmospheric pressures experienced while diving. One retrospective study investigated potential correlations between changes in atmospheric pressure and weather patterns with acute closed-angle glaucoma attacks. One prospective study examined changes in eye parameters before and after SCUBA diving. The final study measured the IOP of subjects wearing a SCUBA diving mask, compared to baseline.

**Table 2:** Characteristics of included studies.

Author (year)	Title	Number of eyes (n)	Number of patients (n)	Age (years) mean (range)	Type of population	Methods	IOP measurement technique	IOP change (mmHg) mean (% reduction)	Pachymetry measured?	CCT at baseline (μm) mean (SD)	Final CCT (μm) mean (SD)
Deleu (2022)	Effect of SCUBA diving on ophthalmic parameters	15	15	Median age was 48.93 (28–72)	Healthy	IOP measured before diving, 30 and 60 min after a standard deep dive of 25 m depth for 25 min in a dedicated diving pool.	Air tonometry (Nidek Tonoref III)	−2.1 (−12.8%) at 30 min post-dive (*p* < 0.0001). −1.4 (−8.7%) at 60 min post-dive (*p* < 0.0001).	Yes	559.3 (27.8)	30 min post-dive: 566.5 (33.5; *p* = 0.012) 60 min post-dive: 562.9 (28.6; nonsignificant)
Mazo (2023)	Intraocular pressure changes under an atmospheric pressure spectrum in a multiplace hyperbaric chamber	48	24	30.6 (23–40)	Healthy	IOP measured in hyperbaric chamber at 0, 20, 40, 60, 50, 30, and 0 feet, respectively. 10 min spent between each measurement.	Tonopen XL	−1.2 (−8.4%) at 2.8 ATA (60 feet) (*p* = 0.012). Then −2.4 (−16.8%) after rising to 1.91 ATA (30 feet) (*p* < 0.001). Then −1.2 (−8.4%) when ending at 1 ATA (0 feet) (*p* = 0.012).	Yes	543.7 (30.4)	N/A
Van de Veire (2008)	Influences of atmospheric pressure and temperature on intraocular pressure	54	27	23.8 (18–44)	Healthy	IOP measured in hyperbaric chamber at 1 and 2 bar at 24 and 28 degrees celsius. It took 10 min to switch bar, 10–20 min for temperature change, and 5 min were given after each change for acclimatization.	Perkins applanation tonometry	Right eye: −1.1 (−9.3%) at 2 bar (*p* = 0.024). Left eye: −1.4 (−12.0%) at 2 bar (0.0006).	Yes	Not mentioned	N/A
Vercellin (2021)	Agreement of rebound and applanation tonometry intraocular pressure measurements during atmospheric pressure change	12	12	43.6	Healthy	IOP measured in hyperbaric chamber with rebound and applanation tonometry at 1, 2, 3, and 4 bar during compression and 3 and 2 bar during decompression. 5 min rest period at each pressure.	Icare rebound tonometer Perkins applanation tonometer	Applanation: −3.6 (−26.1%) at 4 bar (p < 0.0001). Rebound: −3 (−19.6%) at 4 bar (*p* < 0.0001).	Yes	587.4 (12.5)	N/A
Albis-Donado (2020)	Effects of acute atmospheric pressure changes on dynamic contour tonometry and Goldmann applanation tonometry in normal individuals: a pilot study	44	22	33.4 (25–62)	Healthy	IOP measured with GAT and DCT in hyperbaric chamber at 1, 1.1, 1.2, and 1.25 QATM. Measurements taken after 5 min at each pressure.	Goldmann applanation tonometry Dynamic contour tonometry	GAT: +0.16 (+1.3%) at 1.25 QATM. DCT: −1.5 (−8.9%) at 1.25 QATM.	No	Not mentioned	N/A
Huang (2002)	Refractive change in response to acute hyperbaric stress in refractive surgery patients	20	10	52 (46–57) in RK group, 47 (45–48) in LASIK group, and 38 (21–48) in the control group.	6 eyes with previous RK. 5 eyes with previous LASIK. 9 healthy control eyes.	IOP measured before, during, and after spending 15 min at 4 ATM in a hyperbaric chamber.	Tonopen XL	No significant change in IOP for any group.	Yes	RK group: 564 (43) LASIK group: 501 (38) Control group: 528 (17)	RK group: 566 (44) LASIK group: 503 (39) Control group: 527 (16) (nonsignificant)
Peters (1999)	Effect of increased atmospheric pressure on radial keratotomy	8	4	42.5 in control group and 51.5 in RK group	4 eyes with previous RK. 4 healthy control eyes.	IOP measured at baseline and in a hyperbaric chamber after 1 h at 3 ATM.	Unspecified	No change in mean IOP for either group.	Yes	Not mentioned	N/A
Goenadi (2016)	The effect of a diving mask on intraocular pressure in a healthy population	40	20	29.7	Healthy	IOP measured at baseline and with a diving mask (lens removed).	AVIA Tonopen	−0.4 (−2.5%) with mask on (*p* < 0.05).	Yes	544.4 (43.5)	N/A
Bojić (2001)	Acute angle-closed glaucoma and meteorological factors in split, croatia	73	73	57 women with mean age of 67.8 and 16 men with mean age 64.9.	Eyes with acute closed angle glaucoma.	Atmospheric pressure, sunshine hours, and air temperature data were correlated with incidence of acute closed angle glaucoma attacks.	N/A	N/A	N/A	N/A	N/A

IOP, intraocular pressure; CCT, central corneal thickness; SD, standard deviation; ATA, atmosphere absolute; QATM, Queretaro atmospheric pressure; ATM, atmospheric pressure.

### IOP before and after SCUBA diving

Deleu et al. was the only study reporting IOP readings measured pre- and post-diving in a healthy Caucasian population. Their results demonstrated a decrease in mean IOP from 16.4 ± 2.0 mmHg to 14.3 ± 2.3 mmHg (88.1%; *p* < 0.0001), measured 30 min after a 5-min dive to 25 meters, with the Nidek Tonoref III (NIDEK Co., Ltd., Tokyo, Japan) (23). This effect was found to be persistent, with IOP at the 60-min post-dive timepoint continuing to remain low compared to baseline, at 15.0 ± 2.7 mmHg (91.4%; *p* < 0.0001).

### Hyperbaric chamber studies

Various articles studied the results of a high-pressure environment on IOP in a controlled setting within the hyperbaric chamber. Mazo et al. reported findings in a healthy population of students and instructors at the National Navy Diving and Rescue School in a multiplace hyperbaric chamber (24). IOP measurements were taken using a Tonopen XL (Reichert, Depew, New York, United States) at 10 min intervals. Measurements were recorded during simulated descent every 20 feet until reaching 2.8 absolute atmospheric pressure (ATA) or 60 feet, and during simulated ascension at 50 and 30 feet. The mean adjusted IOP, adjusted for central corneal pachymetry, was found to decrease from 14.3 ± 2.2 mmHg to 13.1 ± 2.6 mmHg (*p* = 0.012) as the participants pressurized from sea level to 60 feet (2.8 ATA). During the depressurization phase to 30 feet, the mean adjusted IOP continued to decrease to 11.9 ± 2.7 mmHg (*p* < 0.001) and rose to 13.1 ± 2.9 mmHg (*p* = 0.012) by the end of the session, demonstrating a residual effect on IOP even after returning to lower atmospheric pressures.

Van De Veire et al. reported similar results in 27 healthy volunteers in a hyperbaric chamber, with the use of a Perkins applanation tonometer (Clement-Clarke International, Harlow, Essex, United Kingdom) (25). Mean IOP was found to decrease significantly from 11.8 mmHg to 10.7 mmHg in the right eye (*p* = 0.024) and 11.7 mmHg to 10.3 mmHg in the left eye (*p* = 0.0006) when pressure was increased from 1 to 2 bars (equal to conditions experienced during underwater diving at 10 m). The IOP remained low during the period of the atmospheric pressure increase over 40 min and was independent of temperature change. Additionally, IOP was found to remain decreased from baseline after completion of the hyperbaric cycle (60 min), with the IOP in the right eye reducing from 11.8 mmHg to 11.0 mmHg and left eye from 11.7 mmHg to 11.2 mmHg, although these changes did not reach statistical significance.

In addition to studying the absolute change in IOP in a high-pressure environment, two studies also compared measurements between IOP measuring devices. Vercellin et al. performed a comparison study between the Perkins applanation tonometry and Icare rebound tonometry (Icare, Tiolat Oy, Helsinki, Finland) on 12 eyes from 12 healthy volunteers (26). Measurements were taken with each type of tonometer on a different eye on all patients in a hyperbaric chamber at 1, 2, 3, and 4 bar during the compression phase and at 3 and 2 bar during the decompression phase. An acclimatization period of 5 min was given before each measurement. IOP measured with applanation tonometry had a mean baseline value of 13.8 ± 2.6 mmHg, decreasing to 10.2 ± 1.9 mmHg at 4 ATM (*p* < 0.0001). Rebound tonometry measured a mean baseline IOP of 15.3 ± 2.5 mmHg that decreased to 12.3 ± 1.7 mmHg at 4 ATM (*p* < 0.0001). IOP increased toward baseline with both tonometers during decompression. The difference between IOP measurements with each tonometer remained constant at each measurement.

Albis-Donado et al. also compared the difference in IOP measurements between two IOP measurement devices—the Goldmann applanation tonometry (GAT) and dynamic contour tonometry (DCT), within a hyperbaric chamber (27). Measurements were taken with both tonometers in 44 eyes from 22 healthy volunteers at 1, 1.1, 1.2, and 1.25 Queretaro Atmospheric Pressure (QATM). An acclimatization period of 5 min was given after each pressure change, prior to IOP measurement. The mean IOP measured with the GAT at 1 QATM was 12.2 ± 2.84 mmHg and measured 11.1 ± 2.8, 11.1 ± 2.5, 12.4 ± 3.1 mmHg at 1.1, 1.2, and 1.25 QATM, respectively. With the DCT, IOP was 16.4 ± 2.8 mmHg at 1 QATM and decreased to 15.6 ± 3.0, 15.4 ± 2.9, and 14.9 ± 2.7 mmHg at 1.1, 1.2, and1.25 QATM, respectively.

Two studies examined the relationship between high-pressure environments and IOP in post-operative patients. Huang et al. conducted a prospective study to quantify the change in visual acuity and IOP with increased atmospheric pressure in eyes that underwent refractive surgery, compared to healthy control eyes with no prior refractive surgery (28). The study cohort included 6 eyes that had undergone radial keratotomy (RK), 5 eyes that had undergone myopic laser *in situ* keratomileusis (LASIK), and 9 control eyes. IOP was measured with Tonopen XL before, during, and after exposure to 4 ATM for 15 min in a hyperbaric chamber. No significant differences in IOP between the post-surgical and control groups were observed, leading authors to suggest that recreational diving could be safe post-RK or LASIK, although the study power was limited given the small number of participants. Similar findings were demonstrated by Peters et al., who compared visual acuity and IOP between 4 eyes which underwent previous RK against 4 control eyes matched for age and sex. Measurements were taken immediately before and after hyperbaric chamber exposure to a pressure of 3 ATM for 1 h (29). No significant change in mean IOP was found in either group.

### Atmospheric pressure and angle closure glaucoma

Low light environments have been reported to increase the risk of acute angle closure glaucoma through causing physiological mydriasis (30). To explore this relationship, Bojićc et al. performed a retrospective study into the relationship between weather and meteorological factors and the incidence of acute angle closure glaucoma on 73 cases in Croatia (31). However, no significant correlation between atmospheric pressure and incidence rate of acute closed angle glaucoma was observed.

### Impact of diving mask on IOP

To provide a watertight seal while diving, diving masks need to fit tightly against the periorbital region, with the potential risk of raising the IOP. Goenadi et al. explored this possible relationship by studying the effect of dive mask wearing on IOP, in a cohort of 40 eyes from 20 healthy volunteers (32). The AVIA Tono-Pen (Reichert Inc., NY, United States) was used to measure IOP at baseline with and without the diving mask (with the lenses removed) worn. Contrary to expectations, the mean IOP at baseline was 17.23 ± 2.18 mmHg, which decreased by 0.43 mmHg (*p* < 0.05) to 16.80 ± 2.57 mm Hg with the diving mask on.

## Discussion

The studies included in this review largely demonstrated a decrease in IOP with increased atmospheric pressure, whether in a hyperbaric chamber or in an actual diving environment. Only two studies, Huang et al. (28) and Peters et al. (29) showed inconclusive results. This discrepancy may be attributed to the smaller sample sizes in these studies compared to others. Nonetheless, none of the studies in this review demonstrated an increase in IOP while diving.

The relationship between IOP and hyperbaric environments may be a result of several physiological mechanisms relating to changes in the partial pressure of oxygen and ocular blood flow, as well as other factors including exercise, temperature, and equipment. High atmospheric pressures result in peripheral vascular constriction, which in turn results in high ocular perfusion pressures, which may decrease IOP (13, 14). Changes in the partial pressure of oxygen may also cause vasoconstriction, which decreases IOP by decreasing choroidal blood volume (18), as demonstrated in animal models and human experiments (17–20). In hyperbaric chambers, at high atmospheric pressures the partial pressure of oxygen is also increased, hence resulting in a decrease in IOP.

In addition to environmental pressure and oxygenation, temperature may also influence IOP during diving. A recent study by Hartmann et al. showed a significant relationship between lower temperatures and increased IOP (33). This was attributed at least partly to higher systolic blood pressures, which increase with lower temperatures. Among the studies included in this review, only Van de Veire et al. (25) investigated the impact of temperature change on IOP. The study found a small decrease in IOP when temperatures were reduced from 28°C back to baseline (24°C), with the atmospheric pressure kept constant. However, this result did not reach statistical significance. Bojić et al. (31) revealed a significant correlation with winter months and the incidence of acute angle closure glaucoma, even though no correlation was found for hours of sunshine, air temperature and atmospheric pressure. This relationship was further supported by Zhu et al. who reported a rise in incidence of acute angle closure glaucoma in colder temperatures (34). This may be a result of mechanical or anatomical changes in the peripheral iris and angle structures (35), thus as divers are exposed to cold temperatures during their course of activity, predisposed individuals may possibly be also at a greater risk for acute angle closure glaucoma.

Exercise may also be a confounding variable that influences IOP during SCUBA diving, an activity that requires both endurance and strength, hence demanding significant energy expenditure (36). The relationship between exercise and IOP reduction has been well established (37–39), with this effect observed to be transient and directly correlating with the intensity of exercise (40). In the study by Deleu et al. (23), subjects underwent a dive for 25 min and authors postulated that the IOP reduction in their study may have been due to the sympathetic response triggered by exercise. An exercise-induced sympathetic response is accompanied by β2-adrenergic receptor activation, which is thought to contribute to increased aqueous humor outflow through an increase in trabecular meshwork thickness, along with expansion of both the area and perimeter of Schlemm’s canal (41). Sympathetic activation also causes vasoconstriction of choroidal vasculature, which decreases choroidal blood flow and IOP. Other theories involve the physiological impact of exercise on blood parameters, including a decrease in blood pH, elevation of plasma osmolarity, and an increase in blood lactate levels, although the mechanisms behind these parameters and their influence on IOP remain poorly understood (40).

It should be noted that the use of different IOP measuring techniques and devices may affect IOP readings in environments with different atmospheric pressures. Albis-Donado et al. (27) reported increasing differences in IOP measurements between Goldmann applanation tonometry (GAT) and dynamic contour tonometry (DCT) with lower atmospheric pressures, with the difference in readings increasing by approximately 1 mmHg per 673 m of increased altitude above sea level. Authors concluded that the GAT may not adjust to changes in atmospheric pressure as well as DCT and hence the GAT may be less reliable at higher atmospheric pressures. The authors elaborated that DCT is able to calibrate itself by performing zeroing based on atmospheric pressure, whereas the results of applanation tonometry will be affected by changes in the absolute pressure and resistance to applanation when atmospheric pressure fluctuates (42). In contrast, Vercellin et al. (26) reported agreement between rebound and applanation tonometry IOP measurements with compression up to 4 bar, suggesting that rebound tonometry may be viable for assessing IOP within certain atmospheric pressure ranges.

Corneal thickness is another factor that has been studied in hyperbaric environments. Central corneal thickness (CCT) measurements have been known to confound tonometry readings, particularly when taken with the GAT (43, 44). Of the studies included in this review, only Mazo et al. (24) corrected IOP for CCT. Some studies (25, 28), demonstrated no significant changes in CCT during or after hyperbaric exposure, as well as with increased atmospheric pressure. Goenadi et al. (32) also did not find a correlation between CCT and IOP changes upon wearing of a diving mask. However, other studies have demonstrated CCT changes after exposure to hyperbaric environments, including Peters et al. (29), who reported slight corneal thinning following hyperbaric exposure, and Deleu et al. (23), who reported a statistically significant increase in mean CCT 30-min post-dive, which resolved 30-min later.

The use of diving masks may be expected to result in an increase in IOP due to mechanical compression of the orbits and the subsequent increase in episcleral venous pressure. However, Goenadi et al. (32) reported a small but statistically significant decrease in IOP when subjects donned a diving mask. While swimming goggles are known to elevate IOP (45), authors suggested that the larger frame of the diving mask likely allowed for a more extensive area of the mask to sit on the bony orbit, hence mitigating the pressure exerted on the periocular soft tissue and transmitted to the globe. Another study by Islam et al. did not demonstrate any significant change in IOP when subjects donned a diving dry suit (46).

This systematic review has revealed certain gaps in literature. First, none of the studies included in this review examined subjects exposed for more than 1 h at increased atmospheric pressure, nor measured the impact following repeated dives—which have been found to have subclinical ophthalmological effects (47). Second, structural and functional tests in the form of visual field testing or retinal nerve fiber layer thickness examination were not explored in any of the studies. These additional parameters may be useful in providing a better understanding of the effects of these environments on IOP, retinal nerve fiber layer health, and glaucoma. Third, likely due to feasibility and logistical considerations, most studies utilized hyperbaric chambers in their experiments. These conditions may not fully replicate the actual underwater environments experienced by divers. Fourth, the subjects included in all experiments in this review had healthy eyes which limits its applicability to glaucomatous eyes, which may exhibit a pathological response to environmental changes (48).

Limitations of this review include variations in atmospheric pressures, time intervals for measurements, and tonometry methods among the included studies. This high degree of heterogeneity among the studies may limit generalizability of the conclusions from this review. While studies have explored the relationship between hyperbaric environments and IOP, the precise mechanisms underlying these observations and findings remain poorly understood. More longitudinal studies performed under actual diving environmental conditions may be considered to more accurately assess the effect of diving on IOP and glaucoma.

Nonetheless, to the best knowledge of the authors, this systematic review is the first of its kind to consolidate current literature on the relationship between diving, hyperbaric environments and IOP. As diving becomes increasingly popular and the prevalence of glaucoma continues to rise, information in this niche area of ophthalmology and underwater medicine will become more valuable in the clinical care of divers with, or at risk of glaucoma.

## Data availability statement

The original contributions presented in the study are included in the article/supplementary material, further inquiries can be directed to the corresponding authors.

## Author contributions

PL: Conceptualization, Data curation, Formal analysis, Funding acquisition, Investigation, Methodology, Project administration, Resources, Software, Supervision, Validation, Visualization, Writing – original draft, Writing – review & editing. SL: Conceptualization, Data curation, Formal analysis, Funding acquisition, Investigation, Methodology, Project administration, Resources, Software, Supervision, Validation, Visualization, Writing – original draft, Writing – review & editing. BB: Conceptualization, Investigation, Methodology, Writing – original draft, Writing – review & editing. DM: Writing – review & editing. SD: Writing – review & editing. BA: Conceptualization, Data curation, Formal analysis, Funding acquisition, Investigation, Methodology, Project administration, Resources, Software, Supervision, Validation, Visualization, Writing – original draft, Writing – review & editing.
